# Prevalence and clustering of cardiovascular disease risk factors in rural Nepalese population aged 40–80 years

**DOI:** 10.1186/s12889-018-5600-9

**Published:** 2018-05-31

**Authors:** Mahesh Kumar Khanal, M. S. A. Mansur Ahmed, Mohammad Moniruzzaman, Palash Chandra Banik, Raja Ram Dhungana, Pratiksha Bhandari, Surya Devkota, Arun Shayami

**Affiliations:** 1 Bangladesh Institute of Health Sciences (BIHS), Dhaka, Bangladesh; 2 Department of Community Medicine, Bangladesh Institute of Health Sciences (BIHS), Dhaka, Bangladesh; 3 Padmakanya Campus, Tribhuvan University (TU), Kathmandu, Nepal; 4 Institute of Medicine (IOM), Tribhuvan University, Kathmandu, Nepal; 5 Ministry of Health and Population (MoHP), Kathmandu, Nepal; 6 College of Health and Biomedicine, Victoria University, Melbourne, Australia

**Keywords:** Cardiovascular diseases, Risk factors, Clustering of CVD risk factors, Smoking, Hypertension, Diabetes mellitus, Dyslipidemia, Rural population, Nepal

## Abstract

**Background:**

Cardiovascular diseases (CVD) are the main cause of mortality in low- and middle-income countries like Nepal. Different risk factors usually cluster and interact multiplicatively to increase the risk of developing acute cardiovascular events; however, information related to clustering of CVD risk factors is scarce in Nepal. Therefore, we aimed to determine the prevalence of CVD risk factors with a focus on their clustering pattern in a rural Nepalese population.

**Methods:**

A community-based cross-sectional study was conducted among residents aged 40 to 80 years in Lamjung District of Nepal in 2014. A clustered sampling technique was used in steps. At first, four out of 18 wards were chosen at random. Then, one person per household was selected randomly (*n* = 388). WHO STEPS questionnaires (version 2.2) were used to collect data. Chi-square and independent t-test were used to test significance at the level of *p* < 0.05.

**Results:**

A total 345 samples with complete data were analyzed. Smoking [24.1% (95% CI: 19.5–28.6)], harmful use of alcohol [10.7% (7.4–13.9)], insufficient intake of fruit and vegetable [72% (67.1–76.6)], low physical activity [10.1% (6.9–13.2)], overweight and obesity [59.4% (54.2–64.5)], hypertension [42.9% (37.6–48.1)], diabetes [16.2% (14.0–18.3)], and dyslipidemia [56.0% (53.0–58.7)] were common risk factors among the study population. Overall, 98.2% had at least one risk factor, while 2.0% exhibited six risk factors. Overall, more than a half (63.4%) of participants had at least three risk factors (male: 69.4%, female: 58.5%). Age [OR: 2.3 (95% CI: 1.13–4.72)] and caste/ethnicity [2.0 (95% CI: 1.28–3.43)] were significantly associated with clustering of at least three risk factors.

**Conclusions:**

Cardiovascular risk factors and their clustering were common in the rural population of Nepal. Therefore, comprehensive interventions against all risk factors should be immediately planned and implemented to reduce the future burden of CVD in the rural population of Nepal.

## Background

Cardiovascular diseases (CVD) are the number one cause of mortality globally. In 2015, global estimated deaths caused by CVD were 17.7 million. More than three-quaters of these deaths occur in low- and middle-income countries [1]. In Nepal, the estimated age-standardized death rates caused by CVD (Ischemic Heart Disease and Cerebrovascular Diseases) were 152 and 82 per 100,000 population respectively in 2008 [2]. CVD were the second most common (40.0%) noncommunicable diseases among indoor patients of the non-specialist hospitals of Nepal in 2010 [3]. Moreover, 13.8% of industrial workers of Nepal were diagnosed with CVD in 2016 [4].

Based on attributable deaths globally, common CVD risk factors are high blood pressure (to which 13.0% of global deaths is attributed), tobacco use (9.0%), diabetes (6.0%), physical inactivity (6.0%), overweight/obesity (5.0%), cholesterol (4.5%), harmful use of alcohol (4.0%), and low fruit and vegetable intake (3.0%) [2, 5, 6]. In Nepal, hypertension was the most prevalent risk factor for CVD which ranged from 26.0% to 38.9% [7–11] during the last 3 years. Moreover, diabetes mellitus was seen in 8.4% of Nepalese population [12]. STEPS survey of Nepal in 2013 detected hypercholesterol in 23.0%, smoking in 19.0%, overweight in 21.0%, raised blood glucose in 4.0%, physical inactivity in 3.0%, and harmful use of alcohol in 2.0% [7]. These behavioral and metabolic risk factors usually cluster together, interact, and multiply so that the total risk of developing acute cardiovascular events is increased [13, 14]. In Asia, for instance, almost 44.0% of Chinese adult population had clustered at least two cardiovascular risk factors [15]. Moreover, in Nepal, more than 60.0% had a minimum of two clustered risk factors [7]. Evidence shows that about 58.0% decline in CVD mortality has been attributed to reductions in the population levels of these risk factors [16, 17].

A recent study from China reported that low-income areas had a higher prevalence of total CVD compared to high-income areas [18]. In addition, cardiovascular health is poor in rural communities and disproportionally affects elderly populations [19]. Risk factors and their clustering phenomenon should be explored to plan and organize individual and population-based interventions in rural communities focusing on elderly population. However, there is no data available on status and clustering of all cardiovascular risk factors in the elderly rural population in Nepal. Therefore, we aimed to determine the prevalence of CVD risk factors with a focus on their clustering in rural Nepalese population aged 40–80 years.

## Methods

### Study design, settings, and participants

The current article is a part of the study titled “Risk prediction of cardiovascular disease in selected rural communities of Nepal”, details of the study are available elsewhere [20]. Briefly, the original study was a community-based cross-sectional study carried out in two rural villages (Bhotewodar and Sunderbazar) of Lamjung district (about 200 km west from capital city Kathmandu) of Nepal from October to November in 2014. The study area had 3869 households where 14,275 people were residing. Of these, about one-quater (26.0%) were aged 40 to 80 years in the study area. Indigenous people like Gurung, Tamang, Magar, and Newar were major inhabitants in addition to Bramhan and Kshetri in the study site. Most of them were involved in agricultural work, livestock activities, grocery shops, and locally available jobs [21]. There were two sub-health posts and two fifteen-bed hospital in the communities. Any person who was residing for more than 6 months, aged 40–80 years, and without a history of self-reported angina, myocardial infarction, stroke, intermittent claudication were included in the study. Those with an intelectual disability, bed-ridden patients, and pregnant women were excluded from the study.

### Sample size and sampling

The estimated sample size was calculated based on the prevalence of current smoking (19.0%) reported by nationwide study of Aryal et al. [7] at 5% of allowable error. After adjusting for finite population correction and design effect (1.5), minimum required sample size was 331. With an expectation of 15% non-response rate, the sample size was inflated to 390. The study used a clustered sampling technique in three stages. The study considered the ward as the primary cluster which is the geographic and administrative unit of the communities. Culturally, economically, geographically, and socially diverse populations were residing in each ward, and these features were similar between wards [21]. The wards were selected based on per probability proportion to size (PPS). The sample size for each cluster was determined based upon their population size. In the first stage, out of 18 wards, four were selected. Secondly, systematic random sampling technique was used to select the required number of households from each ward. Every sixth household was considered for the study, starting from an arbitrary point. Finally, we used age and gender of the eligible participants of that household to place in selection grid of the Kish table so that we would interview only an eligible participant from each household [22].

### Data collection

Data was collected by comprehensively trained health workers (two Auxiliary Nurse Midwifes, one Auxiliary Health Worker, and two third-year medical students) using standard WHO STEPS instrument version 2.2 [22]. We adopted all the core questionnaires as well as few of the extended questionnaires in our final tool. The tool was translated into Nepali by applying the forward and backward methodology and then pretested to 20 participants before the final implementation for piloting. Information on socio-demographic condition, behavioral risk factors, such as tobacco use, alcohol use, fruit and vegetable intake, physical activity, history of blood pressure, and diabetes were obtained by asking related questionnaires. For instance, we asked the question “do you currently smoke any tobacco products, such as cigarettes, cigars or pipes” to acquire information on current tobacco use. Alcohol consumption habit was ascertained asking “have you consumed an alcoholic drink within the past 30 days”. If yes, we used show cards to estimate an average amount of standard drinks per day. Similarly, for assessing fruit and vegetable intake, we asked the question, “on how many days do you eat fruit and vegetable in a typical week”. If they responded one or more days, we displayed show cards to calculate average numbers of fruit and vegetable servings per day. Height, weight, waist circumference, and blood pressure were measured precisely using standard methods as described in WHO STEPs manual [22]. Height and weight were measured over a light cloth and without foot bearings using the portable weighing machine and stadiometer, respectively. The non-elastic measuring tape was used to measure waist circumference at the level of the umbilicus (midway between last rib and anterior superior iliac spine) during normal respiration. Doctor’s aneroid sphygmomanometer with an appropriate sized cuff and a stethoscope were utilized over the unclothed left arm to measure blood pressure. Three blood pressure readings were obtained, the first after at least 15 min, second and third after at least five-minute intervals. Finally, the participants were invited in the next morning, after overnight fasting, to a nearby hospital or community centre, where, skilled phlebotomist withdrew venous blood to measure fasting blood sugar and lipid profile. Participants without known diabetes were then requested to drink 75 g of anhydrous glucose mixed with 250 ml of water within five minutes. Another one milliliter of venous blood was also collected two hours after the oral glucose intake for oral glucose tolerance test. Blood sugar and lipid profile were measured using a semi-automated machine of Erba Mannheim (Germany), chem. 5 v3 model in the laboratory of a nearby community hospital.

### Definition of variables

#### Demographic and socio-economic variables

Age was taken in complete years. Education was categorized into illiterate and no formal education (< grade 1), primary (grade 1–5) and more than or equal to secondary (≥ grade 6). Caste was divided into upper caste (Bramhan and Kshetri), Janajati (Gurung, Tamang, Newar, Magar, and Thakali) and Dalit (low caste). Information related to occupation was subdivided into employed, self-employed (operating own business), unemployed (student or house-maker or non-paid workers), and retired. Poor was defined as any participant whose family income per person per year was less than 18,428 rupees ($184.2) [23].

#### Variables related to behavior (STEP I)

Current smoking was defined as those who were smoking cigarettes and those who quit less than 1 month before data collection. Similarly, those who were chewing tobacco in the last 30 days were defined as current smokeless tobacco users [22]. The total amount of alcohol intake was calculated in a number of the standard drink (10 g of pure ethanol). Average consumption of alcohol of at least one (women) or two (men) standard drinks per day over last 30 days was defined as the harmful use of alcohol [24]. Insufficient intake of fruit and vegetable was considered if the participants consumed less than five servings (400 g) per day [22]. Physical activities were measured in metabolic equivalents of task (METs) minutes per week. Low level physical activities (physically inactive) was defined as less than 600 MET-minutes per week of physical activities [22].

#### Anthropometric measurements (STEP II)

Weight was divided by square of height (in meters) to calculate Body Mass Index (BMI) of participants. The BMI was classified as underweight (< 18.5 Kg/m^2^), normal (18.5–24.9 Kg/m^2^), overweight (≥25–29.9 Kg/m^2^), and obese (≥30 Kg/m^2^) [25]. Raised waist circumference (central obesity) was defined as the raised level if it was more than 88 cm in women and more than 102 cm in men [26]. For blood pressure, an average of the last two readings was used in the final analysis. Hypertension was defined as average systolic blood pressure (SBP) ≥ 140 mmHg and/or average diastolic blood pressure (DBP) ≥ 90 mmHg and/or history of taking antihypertensive medication in the last 2 weeks [27].

#### Biochemical variables (STEP III)

Diabetes mellitus was defined by the presence of fasting blood sugar ≥126 mg/dl (milligram/deciliter) and/or post prandial blood sugar ≥200 mg/dl and/or intake of any anti-diabetic drugs [28]. Dyslipidemia was defined as presence of at least one of the following; raised total cholesterol (> 200 mg/dl), raised triglyceride (> 150 mg/dl), raised low-density lipoprotein (> 130 mg/dl), decreased high-density lipoprotein (< 40 mg/dl in male and < 50 mg/dl in female), and/or use of antilipidemic drug [29].

#### Clustering of risk factors

The clustering of modifiable CVD risk factors were assessed based on the presence of eight major risk factors; current smoking, harmful use of alcohol, low fruit and vegetable intake, low physical activity, overweight or obesity, hypertension, diabetes, and dyslipidemia.

### Statistical analysis

Data was compiled and edited to maintain consistency before entering into Epidata version 3.1. Duplications were removed and exported into SPSS V.16.0 for further analysis. Simple descriptive statistics were used for socio-demographic characteristics. Prevalence of risk factors was computed in percentage with 95% confidence interval (CI). In the next step, age and sex adjusted prevalence of CVD risk factors was computed using WHO world standard population distribution between 2000 and 2025 considering sex weight of 0.5 [30]. Clustering of risk factors was presented as a percentage. Mean and standard deviations (SD) were reported for normally distributed continuous variables. Median with interquartile range was reported for non-normally distributed continuous variable. Correlation between different risk factors was determined using Pearson’s correlation coefficients. Socio-demographic variables were entered into simple and multiple logistic regression models to determine crude and adjusted odds ratios, respectively. Chi-square and independent t-tests were used to compare categorical and continuous variables, respectively. Mann-Whitney U test was used to test for non-normally distributed variables. All tests were two tail test and *p* < 0.05 was considered statistically significant.

### Ethical consideration

Ethical Review Board of Nepal Health Research Council reviewed and approved the study protocol. Before data collection, data enumerator informed all the eligible participants about the study objectives, data collection procedures, benefits and risks of the study, confidentiality, and anticipated use of the results. Then, the enumerators (health professionals) obtained written consent or thumb impression (if unable to write) using a consent form in Nepali language.

## Results

### Socio-demographic characteristics

Overall, 345 samples with complete data were analysed excluding 43 participants who did not participate in biochemical assessment. Socio-demographic characteristics of the study subjects are presented elsewhere [20]. In brief, more than half of the participants were females (55.4%), aged 53.5 ± 10.1 years, and 40% of them were aged 40–49 years. About half of the participants (53.6%) were uneducated and from Janajati (55.5%) population. The majority were unemployed (58.0%). Almost all participants were married (91.3%) and poor (97.1%). Out of participants who did not attend the biochemical assessment, mean age was 52 ± 10.7 years, 55.8% were female, 55.8% did not have formal education, 51.2% were Janajati, 67.4% were unemployed. These characteristics were not significantly different from the participants who had completed the study.

### Prevalence of cardiovascular disease risk factors

On average, participants were smoking at least two cigarettes per day. Additionally, participants were consuming at least one standard drink per day. Overall, the median intake of fruit and vegetable was four servings per day, and median physical activity was 3360 METs-minutes per week. Mean body mass index was 25.9 (± 4.2) Kg/m^2^ and mean waist circumference was 88.8 (± 11.7) cm. The average systolic and diastolic blood pressure were 124.5 (± 18.7) mm Hg and 88.8 (± 10.8) mm Hg, respectively. The mean fasting blood sugar, total cholesterol, triglyceride, high-density lipoprotein, and low-density lipoprotein were 92.8 (± 34.1) mg/dl, 165.1 (± 34.9) mg/dl, 129.9 (± 69.2) mg/dl, 53.2 (± 12.5) mg/dl, and 86.3 (± 33.6) mg/dl, respectively. Standard drinks of alcohol per day (*p* = < 0.001), waist circumference (*p* = < 0.001), diastolic blood pressure (*p* = 0.008), total cholesterol (*p* = 0.009), and triglyceride (*p* = 0.009) were significantly different between men and women (Table 1).

**Table 1:** Distribution of cardiovascular disease risk factors of study subjects by gender

Risk factors	Both sexes	Men	Women	*P* value
	Mean (SD)	Mean (SD)	Mean (SD)	
Average cigar per day	1.98 (5.2)	2.2 (5.7)	1.8 (4.7)	0.478
Standard drinks of alcohol/day	0.8 (2.5)	1.6 (3.5)	0.09 (0.64)	< 0.001
Fruit/vegetable (servings/day)^a^	4.0 (2.0)	4 (1.0)	4 (2.0)	0.101
Metabolic equivalent - minute/week ^a^	3360.0 (3576.0)	3150.0 (3600.0)	3600.0 (3525.0)	0.121
Body Mass Index (Kg/m^2^)	25.9 (4.2)	25.9 (3.4)	25.9 (4.6)	0.996
Waist circumference (cm)	88.8 (11.7)	91.9 (9.9)	86.3 (12.3)	< 0.001
Systolic Blood Pressure (mmHg)	124.5 (18.7)	126.8 (18.7)	122.6 (18.4)	0.039
Diastolic Blood pressure (mmHg)	79.9 (10.8)	81.6 (11.6)	78.5 (9.9)	0.008
Fasting Blood Sugar (mg/dl)	92.8 (34.1)	91.7 (23.8)	93.6 (40.5)	0.61
Total cholesterol (mg/dl)	165.1 (34.9)	170.6 (34.4)	160.7 (34.8)	0.009
Triglyceride (mg/dl)	129.9 (69.2)	138.7 (76.6)	119.2 (61.4)	0.009
High-density Lipoprotein (mg/dl)	53.2 (12.5)	53 (12.3)	53.3 (12.7)	0.8
Low-density Lipoprotein (mg/dl)	86.3 (33.6)	89.8 (33.5)	83.4 (33.5)	0.03

^a^*median* Interquartile range, *SD* Standard Deviation

The overall prevalence of current smoking was 24.1% (95% CI: 19.5–28.6) with a higher proportion in men (29.2%) compared to women (20.0%). About one-fifth (19.0%) were using smokeless tobacco (men: 29.6%, women: 11.0%). Overall, 10.7% (95% CI: 7.4–13.9) were drinking a harmful dose of alcohol (men: 21.4%, women: 2.3%). Similarly, 10.1% (95% CI: 6.9–13.2) were physically inactive. Women were less physically active (12.6%) compared to men (7.1%). Additionally, 72.0% (95% CI: 67.1–76.6) were consuming less than five servings of fruit and vegetable daily (men: 76.6%, women: 68.0%) (Table 2).

**Table 2:** Prevalence of cardiovascular disease risk factors stratified by gender and age

Characteristics	Current Smoking	Harmful use of alcohol	Fruit/Vegetable< 5 servings	Inadequate Physical activity	Overweight and obesity	Hypertension	Diabetes mellitus	TC (> 200 mg/dl)	TG(> 150 mg/dl)	HDL (M < 40, F < 50 mg/dl)	LDL(> 130 mg/dl)	Dyslipidemia
Men and Women												
40–49	17.5	9.5	66.4	14.6	64.2	36.5	15.3	19	26.3	32.1	11.7	57.9
50–59	25.7	14.3	76.2	6.7	59.0	42.9	15.1	19	29.5	30.5	9.5	58.1
60–69	31.0	9.9	71.8	8.5	64.8	52.1	15.5	15.5	28.2	23.9	12.7	56.3
70–79	31.3	6.3	81.3	6.3	28.1	50	15.6	6.2	21.9	31.2	3.1	40.6
Total	24.1	10.7	71.9	10.1	59.4	42.9	16.2	17.1	27.2	29.9	10.4	55.9
95% CI	19.5–28.6	7.4–13.9	67.1–76.6	6.9–13.2	54.2–64.5	37.6–48.1	14.0–18.3	14.9–19.2	24.6–29.7	27.2–32.5	8.6–12.1	53.0–58.7
Age sex adjusted prevalence	24.9	10.4	71.5	9.9	58.6	43.2	15.9	16.8	27.5	29.3	10.1	55.4
Men												
40–49	26.2	20.0	75.4	9.2	56.9	44.6	16.9	24.6	35.4	13.8	18.5	55.4
50–59	26.1	28.3	78.3	2.2	69.6	52.2	17.4	17.4	34.8	10.9	8.7	50.0
60–69	37.5	18.8	71.9	12.5	65.6	46.9	15.6	15.6	34.4	18.8	12.5	56.3
70–79	36.4	9.1	90.9	0.0	36.4	36.4	9.1	9.1	27.3	0.0	0.0	27.3
Total	29.2	21.4	76.6	7.1	61.0	46.8	16.2	19.5	34.4	13.0	13.0	51.9
Women												
40–49	9.7	0.0	58.3	19.4	70.8	29.2	13.9	13.9	18.1	48.6	5.6	59.7
50–59	25.4	3.1	74.6	10.2	50.8	35.6	18.6	20.3	25.4	45.8	10.2	64.4
60–69	25.6	4.8	71.8	5.1	64.1	56.4	15.4	15.4	23.1	28.2	12.8	56.4
70–79	28.6	4.2	76.2	9.5	23.8	57.1	19.0	4.8	19	47.6	4.8	47.6
Total	19.9	2.3	68.1	12.6	58.1	39.8	16.2	15.2	21.5	43.5	8.4	59.2
P value (men and women)	0.044	< 0.001	0.079	0.097	0.58	0.194	0.99	0.29	0.007	0.001	0.16	0.18
P value (age groups)	0.105	0.56	0.506	0.224	0.002	0.144	0.94	0.33	0.84	0.66	0.47	0.33

*TC* Total cholesterol, *TG* Triglyceride, *HDL* High-density Lipoprotein, *LDL* Low-density Lipoprotein

Overweight and obesity was observed among 59.4% (95% CI: 54.2–64.5) of participants while 31.3% (95% CI: 26.4–36.1) of participants had central obesity. Overweight and obesity was higher in males (61.0%) than in females (58%), whereas central obesity was higher in women (55.0%) compared to men (14.3%). The overall prevalence of hypertension was 42.9% (95% CI: 37.6–48.1) where more men (46.8%) compared to women (39.8%) had raised blood pressure (Table 2).

Diabetes mellitus was determined in 16.2% (95% CI: 14.0–18.3) of total study subjects. Out of all, dyslipidemia was revealed in 56.0% (95% CI: 53.0–58.7) of participants. Dyslipidemia was higher in women (59.7%) compared to men (52.0%). When analysed separately, 29.9% (95% CI: 27.2–32.5) had raised high-density lipoprotein, 27.2% (95% CI: 24.6–29.7) had elevated triglyceride, 17.1% (95% CI: 14.9–19.2) had increased level of total cholesterol and 10.4% (95% CI: 8.6–12.1) had raised low-density lipoprotein (Table 2).

### Distribution of cardiovascular disease risk factors

The proportion of current smoking (*p* = 0.04), harmful use of alcohol (*p* < 0.001), raised waist circumference (*p* = 0.001), raised triglycerides (*p* = 0.007), and decreased high-density lipoprotein (*p* = 0.001) were significantly different between men and women (Table 2).

The proportion of current smoking, less fruit and vegetable intake, hypertension, and diabetes increased with increased age. In opposition, the proportion of harmful use of alcohol, physical inactivity, overweight/obesity, and dyslipidemia decreased with increased age. However, only overweight and obesity were significantly associated with age (*p* = 0.002) (Table 2).

The harmful use of alcohol was significantly associated with the level of education (*p* = 0.003), caste (*p* = 0.02), and economic status (*p* = 0.046). It was highest among participants who had at least secondary level of education (11.0%) and who were Dalit (28.6%) (Table 3).

**Table 3:** Distribution of cardiovascular disease risk factors by socio-economic characteristics

Characteristics	Current Smoking	Harmful use of alcohol	Fruit/Vegetable< 5 servings	Physical inactivity	Overweight and obesity	Hypertension	Diabetes mellitus	TC (> 200 mg/dl)	TG(> 150 mg/dl)	HDL (M < 40, F < 50 mg/dl)	LDL(> 130 mg/dl)	Dyslipidemia
Level of education												
No formal education	25.9	6.5	73.0	10.8	54.6	41.6	15.1	15.7	23.8	37.8	10.8	56.8
Primary	25.7	21.4	75.7	8.6	67.1	50	8.6	12.9	21.4	18.6	7.1	47.1
≥ Secondary	18.9	11.1	66.7	10	63.3	40	24.4	23.3	38.9	22.2	12.2	61.1
P value	0.41	0.003	0.401	0.868	0.129	0.392	0.02	0.16	0.01	0.002	0.56	0.19
Caste/ ethnicity												
Upper cast	21.8	8.3	72.2	11.3	48.9	39.8	13.5	21.8	28.6	27.1	12	59.4
Dalit	42.9	28.6	76.2	9.5	66.7	52.4	19.0	0.0	14.3	19.0	4.8	33.3
Janajati	23.6	10.5	71.2	9.4	66.0	44.0	17.8	15.7	27.7	33.0	9.9	56.0
P value	0.108	0.02	0.886	0.858	0.007	0.505	0.55	0.03	0.38	0.27	0.56	0.08
Occupation												
Employ	22.5	12.5	67.5	7.5	52.5	30.0	22.5	22.5	42.5	17.5	10.0	65.0
Self employed	16.7	12.8	75.6	14.1	65.4	48.7	7.7	19.2	29.5	29.5	11.5	53.8
Unemployed	26.0	8.0	72.5	10.5	57.0	41.0	17.0	14.5	23.0	35.5	9.5	55.5
Retired	33.3	22.2	63.0	0.0	70.4	59.3	25.9	22.2	29.6	7.4	14.8	51.9
P value	0.254	0.124	0.567	0.193	0.287	0.071	0.06	0.47	0.07	0.006	0.83	0.64
Economic status												
Above poverty line	10.0	30.0	70.0	10.0	50.0	50.0	30.0	20.0	20.0	20.0	10.0	40.0
Poor	24.5	10.1	71.9	10.1	59.7	42.7	31.3	17	27.5	30.1	10.4	56.4
P value	0.291	0.046	0.893	0.988	0.538	0.645	0.58	0.8	0.6	0.49	0.96	0.3

*TC* Total cholesterol, *TG* Triglyceride, *HDL* High-density Lipoprotein, *LDL* Low-density Lipoprotein

Overweight and obesity was associated with ethnicity (*p* = 0.007) with the highest prevalence among Dalit (66.7%) participants. Waist circumference was associated with the level of education (*p* = 0.001) and occupation (*p* < 0.001). It was highest among uneducated (39.5%) and unemployed (38.5%). Hypertension was associated with occupation (*p* = 0.071), and retired participants had highest prevalence (59.3%) (Table 3).

Prevalence of diabetes mellitus was significantly different among different levels of education (p = 0.02). It was highest among those with at least secondary level education (24.4%). Raised total cholesterol (*p* = 0.03) was significantly different among different ethnic groups. Similarly, the raised triglyceride was associated with the level of education (*p* = 0.0 1). High-density lipoprotein was associated with the level of education (*p* = 0.002) and the occupation (*p* = 0.006) (Table 3).

### Correlation between cardiovascular disease risk factors

Correlations between different risk factors were weak. Stronger correlations were observed between smoking and alcohol intake, diastolic blood pressure and body mass index along with waist circumference and triglyceride (Table 4).

**Table 4:** Correlation between different cardiovascular disease risk factors

	CG	AL	FV	MET	BMI	WC	SBP	DBP	FBS	TC	TG	HDL	LDL
CG													
AL	.286^a^												
FV	−.045	−.087											
MET	.018	.023	.016										
BMI	−.096	−.049	.077	−.029									
WC	-.121^b^	−.007	.055	−.090	.569^a^								
SBP	−.010	.117^b^	−.025	.028	.118^b^	.162^a^							
DBP	−.078	.065	−.050	−.065	.201^a^	.219^a^	.769^a^						
FBS	−.013	.011	−.037	−.075	.022	.032	.018	.037					
TC	.010	.100	.037	−.022	−.035	.001	.033	.030	.098				
TG	−.038	−.026	−.012	−.048	.096	.181^a^	.133^b^	.125^b^	.198^a^	.250^a^			
HDL	.078	.173^a^	.040	.012	−.034	−.101	−.005	−.098	.136^b^	.195^a^	−.075		
LDL	−.004	.050	.029	−.008	−.064	−.036	−.019	.016	−.030	.863^a^	-.123^b^	-.140^a^	

*CG* Average no. of cigarettes/day, *AL* Average standard drinks/day, *FV* Total servings of fruit and vegetable/day, *METs* Total METs-Minutes/week, *BMI* Body Mass Index, *WC* Waist circumference, *SBP* Systolic Blood Pressure, *DBP* Diastolic Blood pressure, *FBS* Fasting Blood Sugar, *TC* Total cholesterol, *TG* Triglyceride, *HDL* High-density Lipoprotein, *LDL* Low-density Lipoprotein

^a^Correlation is significant at the 0.01 level

^b^Correlation is significant at the 0.05 level

### Clustering of cardiovascular disease risk factors

**Fig. 1:**
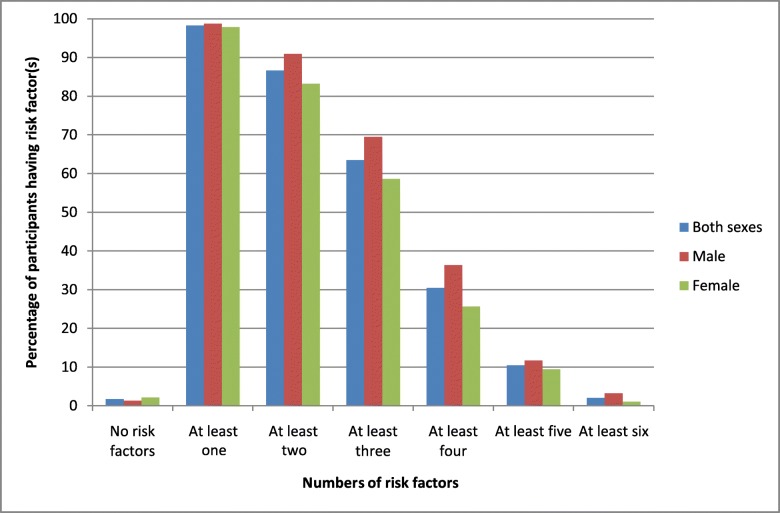
Distribution of clustering of cardiovascular disease risk factors by gender

Almost all of the participants (98.2%) had at least one risk factor. In addition, 86% of total participants had clustering of at least two risk factors. Overall, 63.4% of participants had at least three risk factors (male: 69.4%, female: 58.5%). Clustering of at least four risk factors was observed among aproximately one-third of the participants (30.4%). Clustering was higher among males (36.3%) compared to females (25.6%) (Fig. 1). About 2.0% (male: 3.2%, female: 1%) had up to six risk factors. We studied the socio-economic determinants of clustering of at least three risk factors, as after this clustering was suddenly dropped down. In univariate logistic regression; gender, age, and caste/ethnicity were significantly associated with clustering of at least three risk factors. However, after adjusting for all other socio-demographic factors, age and caste/ethnicity were significantly associated with clustering of risk factors (at least three). Odds of clustering of at least three risk factors was 2.3 times (95% CI: 1.13–4.72) in the age group of 60–69 years compared to 40–49 years of age. Dalits had two times (95% CI: 1.28–3.43) more chance of risk factor clustering compared to upper caste residents (Table 5).

**Table 5:** Socio-demographic factors associated with clustering of cardiovascular risk factors three or more

Characteristics	Crude odds ratio (95% CI)	Adjusted odds ratio (95% CI)
Sex		
Female	Ref^a^	Ref
Male	1.60 (1.02–2.51)	1.53 (0.80–2.93)
Age		
40–49	Ref	Ref
50–59	1.27 (0.75–2.14)	1.39 (0.79–2.45)
60–69	1.89 (1.01–3.53)^a^	2.31 (1.13–4.72)^a^
70–79	0.88 (0.40–1.83)	0.94 (0.38–2.31)
Level of Education		
No formal education	Ref	Ref
Primary	0.78 (0.46–1.33)	0.80 (0.36–1.77)
≥ Secondary	0.95 (0.49–1.85)	0.87 (0.42–1.81)
Caste/Ethnicity		
Upper cast	Ref^a^	Ref^a^
Janajati	0.55 (0.35–0.88)^a^	2.03 (0.71–5.82)
Dalit	1.14 (0.42–3.09)	2.09 (1.28–3.43)^a^
Marital Status		
Married	Ref	Ref
Widow or Widower	0.85 (0.38–1.89)	0.71 (0.29–1.74)
Occupation		
Employ	Ref	Ref
Self employed	1.34 (0.60–3.00)	1.09 (0.46–2.62)
Unemployed	0.93 (0.46–1.89)	0.80 (0.32–2.02)
Retired	1.19 (0.43–3.34)	0.70 (0.22–2.16)
Economic status		
Higher(> NRs 8498)	Ref	Ref
Poor (≤ NRs 8498)	1.16 (0.32–4.20)	1.22 (0.31–4.75)

^a^significant at the 0.05 level

## Discussion

This study determined the prevalence of cardiovascular disease risk factors along with their clustering phenomenon in a rural population of Nepal. Prevalence of each cardiovascular risk factor was high and a maximum of six risk factors was clustered in some study participants.

Our current study revealed that approximately one-quater (24.1%) were smoking cigarettes in a rural population of Nepal. Our finding was consistent with the tobacco smoking prevalence reported in other studies in Nepal. For instance, the proportion of current smoker was 29.0% in STEPS survey 2013 of Nepal among aged 40–69 years [7] and 28.6% in rural Sindhuli [31]. However, the prevalence of smoking found in our study was higher compared to that found in the capital city Kathmandu in urban settings (20.0%) [10] and rural settings (21.7%) [10, 32] and the sub-metropolitan city Pokhara (17.0%) [8] of Nepal. In all of these studies, more males compared to females were smoking cigarettes. These studies revealed the severity of smoking in Nepal.

One in ten study participants was drinking a harmful dose of alcohol in the current study. Similar result was observed in a recent study conducted in Pokhara (12.0%) of Nepal [8]. However, our finding was higher than the report of STEPs survey of 2013 (2.7%) for the 45 to 69 years of age group [7]. Most of the current study subjects were of indigenous and Dalit caste. Such ethnic people were more likely to drink alcohol and brew alcohol at home [33], which might be the reason for higher prevalence.

The burden of low fruit and vegetable intake was high in Nepal. The current study determined that about two-thirds (72.0%) were not consuming five servings of fruit and vegetable per day. This prevalence was lower when compared with the result of a study representing the whole country (99.0%) [7] and a study from surveillance site in Bhaktapur (97.9%) of Nepal [34]. Variation according to age, race/ethnicity, income, education, and availability of fruit and vegetable are established socio-demographic factors for low fruit and vegetable intake [35, 36].

Ten percent of our study participants were engaged in a low level of physical activities. This prevalence was in line with the findings from Pokhara of Nepal (7.0%) and world health survey for physical activities (8.0%) [8, 37]. However, current prevalence was less than the findings of capital city Kathmandu (40.2%) [10], Eastern region (37.6%) [38], and Bhaktapur (male: 18.0% and female: 22.0%) of Nepal [39]. Work and travel related activities are the major domains for physical activities in Nepal [39]. These domains are more pronounced in rural areas, and might be the reason for the lower prevalence of physical inactivity in our study.

About six in ten participants (59.0%) were overweight and obese in our study population. This prevalence was comparable with the result of Western Nepal (60.7%) [38]. However, it was three-times greater than the findings of a stepwise survey of risk factors in Nepal (21.0%) [7]. The higher prevalence of overweight and obesity may be related to the higher proportion of participants who smoke cigarette, chew tobacco, drink alcohol, and indulge in less physical activities in the current study population. Evidences support that alcohol energy intake may be responsible for weight gain if not counterbalanced, for instance, by physical activities [40]. However, further studies would be needed to confirm and explain our findings.

Two-fifths of participants (43.0%) had hypertension in our study. The prevalence of hypertension in the current study (38.9%) was comparable with above-mentioned STEPS survey (47.0%) for the respective age of more than 45 years [7]. However, hypertension was greater in our study compared to populations from Eastern Nepal (34.0%) [38], municipalities of Kathmandu (32.5%) [10], Pokhara-Lekhnath (28.0%) [8], Dhulikhel (27.7%) [9], and Surkhet (38.9%) [11] of Nepal. Alcohol consumption, body mass index, total cholesterol, and triglyceride were correlated to systolic and diastolic blood pressure in our study. The high prevalence of these risk factors in our study might explain the higher rates of hypertension.

The prevalence of diabetes mellitus was 16.2% in our study. This prevalence was higher than the reported prevalences from Eastern Nepal for the age group 40 to 80 years (11.5%) [38], STEPS survey 2013 (9.0%) for age group 45–69 years [7], and rural population of Sunsari (9.0%) [41]. One study conducted in Kathmandu valley reported that 25.9% of participants aged 60 years and older had diabetes mellitus [42]. That means elderly age group has the higher chance of diabetes [43]. A high proportion of diabetes in our study could be a result of the high prevalence of triglyceride that was correlated with fasting blood sugar. Another reason for a higher burden of diabetes in our study may be because of the use of an oral glucose tolerance test for those who did not have known diabetes.

Our study revealed more than half (56.0%) of participants of the rural population had dyslipidemia. This overall dyslipidemia was accounted by 17.0% of elevated total cholesterol, 27.0% of raised triglyceride, 30.0% decreased high-density lipoprotein, and 10.0% of raised low-density lipoprotein among all the study subjects. When compared with above-mentioned STEPS survey, our study population had less prevalence of dyslipidemia. For instance, among participants of age group 45 to 69 years of age, 33% had raised cholesterol, 35% had elevated triglyceride, and 24% had elevated low-density lipoprotein in the STEPS survey [44]. Furthermore, the proportion of dyslipidemia in our study was less compared to another study from non-diabetes participants of the urban area of the capital city Kathmandu, which determined 73.3% had dyslipidemia [45]. Our study revealed that prevalent risk factors namely waist circumference, systolic blood pressure, and fasting blood sugar were correlated with triglyceride level. Similarly, alcohol intake and fasting blood sugar were positively correlated with the reduction of serum high-density lipoprotein. All of these may have been responsible for such high prevalence of dyslipidemia.

Almost all of the study population had at least one risk factor. The finding of our study was consistent with the STEPS survey 2013 of Nepal which revealed 99.6% of participants had at least one risk factor [7]. When compared with the studies from other Asian countries, clustering of at least two risk factors was observed in 44% of Chinese population [15] and 76% of cases in Bangladesh [46]. Comparably, the current study displayed 86% of study participants had a minimum of two risk factors clustered together. The current finding of clustering of three to five risk factors (63.4%) was higher compared to STEPS survey 2013 (30.0%) for the age group 45 to 69 years [7]. Our study included the harmful use of alcohol, diabetes, and dyslipidemia when analyzing the clustering phenomenon, which the STEPS survey did not. This could explain the disparity between the findings.

When different risk factors act together, the effect will be multiplicative and raises the risk of CVD more than the summation of risk factors [13, 14]. For instance, one study reported that incidence of ischemic heart disease rises from 0 to 40% as the number of risk factors conglomerate from zero to five among diabetic patients [47]. Moreover, annual medical expenditure increases with the rise in clustering of risk factors [48]. In our study greatest clustering was observed among 60–69 years of age group and Dalit participant. Therefore, it could be pronounced that preventive strategies should be focused on individuals who have more risk factors and especially elderly population who are aged 60–69 years and are Dalit.

It is important to emphasize health education programs that warn about the behavioral risk factors of CVD. In addition, early detection and treatment of intermediary risk factors (hypertension, diabetes, dyslipidemia and overweight) are required to minimize the future burden of CVD [2]. Health promotion approaches could be delivered through various approaches: health workforce, trained community volunteers of the current health system, school health programs or media campaigns [49–51] collaborating with all stakeholders. Such community-based programs have already been implemented in developed and developing countries [50, 52]. Package of Essential Noncommunicable Disease (PEN) interventions could be implemented to prevent cardiovascular diseases as well, so that the community could utilize current health care delivery system [53]. One study from Northern India revealed that the primary health care setting was a feasible setting for CVD risk management even in rural areas [52].

Our study had several limitations. The study was conducted in a selected rural community where a large population of Janajati and women were residing. Our sample size was not large enough to make it a representative sample of all the risk factors. As we have included participants aged 40 to 80 years, we could not report the status of risk factor among the younger population. The current study might have underestimated the cardiovascular risk factors, as it excluded those who had known heart diseases, stroke, or intermittent claudication. The prevalence of risk factors might have been underestimated as about 11.0% of total participants did not involve in biochemical assessment, though socio-demographic characteristics of responders and non-responders were not significantly different. Despite these limitations, our study had several strengths. This is the first study conducted among elderly rural population of Nepal to explore all traditional risk factors of CVD including biochemical assessment. We used trained enumerators to acquire data related to the participants at their own home. We performed the oral glucose tolerance test to confirm diabetes along with the fasting blood sugar. Therefore, findings could be cautiously generalized to other population.

## Conclusions

Cardiovascular risk factors and their clustering were common in the rural population of Nepal. Almost all of the participants displayed on risk factor, with the majority of the participants presenting with one or clusters of two-three risk factors. When different risk factors aggregate together, their effect would be multiplicative to develop cardiovascular diseases. Therefore, comprehensive intervention to address multiple risk factors should be immediately planned and implemented to reduce the risk factors and future burden of cardiovascular diseases in a rural population of Nepal.

## Abbreviations

BMI: Body mass index
CVD: Cardiovascular diseases
DBP: Diastolic blood pressure
HDL: High-density lipoprotein
LDL: Low-density lipoprotein
METs: Metabolic equivalents of task
NCD: Non-communicable diseases
SBP: Systolic blood pressure
STEPs: Stepwise approach to surveillance
